# Underestimated reason of hyperkalemia in diabetic patients: type IV renal tubular acidosis- mini review

**DOI:** 10.3389/fcdhc.2025.1570868

**Published:** 2025-07-11

**Authors:** Sibel Ertek, Kayser Caglar

**Affiliations:** ^1^ Endocrinology and Metabolic Diseases Department, Losante Hospital, Ankara, Türkiye; ^2^ Nephrology Department, Losante Hospital, Ankara, Türkiye

**Keywords:** diabetes, hyperkalemia, type IV renal tubular acidosis, hyporeninemic hypoaldosteronism, potassium

## Abstract

Diabetes mellitus is chronic disease with increasing prevalence, and may cause many organ complications, including kidneys. Reduced creatinine clearance and kidney failure are important, but hyperkalemia may be present in diabetic patients even before these problems. There may be many reasons of hyperkalemia in this group of patients. Type IV renal tubular acidosis is important cause of generally mild hyperkalemia, and it is a treatable condition. ‘‘Polypharmacy’’ –which is very common in diabetic patients due to accompanying other diseases- may trigger electrolyte imbalance. Underlying causes should be investigated and treatment should be done before it worsens.

## Introduction

Since potassium is largely stored in cells, hyperkalemia is sign of either increased potassium release from cells or decreased urinary output from kidneys (1). Hyperkalemia in chronic kidney disease is usually seen in the advanced stages of nephron loss, but in some cases, especially in diabetic patients, hyperkalemia can be seen in the earlier stages of the disease due to deterioration in tubular functions. Hypoaldosteronism should be considered in patients who develop persistent hyperkalemia without an obvious cause such as renal failure, excessive potassium intake, or use of potassium-sparing drugs (2, 3). Although aldosterone also promotes sodium retention, hypoaldosteronism is not typically associated with prominent sodium wasting because of the compensatory action of other sodium-retaining factors (such as angiotensin II and norepinephrine).

RTA (Renal Tubulary Acidosis) has 3 major forms: distal, proximal, and hyperkalemic RTA. Distal RTA (Type I) is associated with decreased hydrogen ion secretion in the distal nephron, while proximal RTA (Type II) is characterized by impaired bicarbonate (HCO3) reabsorption in the proximal tubules. Hyperkalemic RTA, often called type IV RTA, occurs due to aldosterone deficiency or aldosterone resistance (4).

In type IV renal tubular acidosis, there is selective aldosterone deficiency or aldosterone resistance, as well as decreased ammonia excretion and metabolic acidosis with a normal anion gap (5). Aldosterone increases sodium resorption in exchange for potassium and hydrogen. Aldosterone increases sodium reabsorption in the principal cells of the cortical collecting tubule and makes the lumen electronegative, thus creating an electrical gradient that favors the secretion of potassium into the lumen. Therefore, in aldosterone deficiency or resistance, potassium excretion is impaired and hyperkalemia develops. In most cases these hormones are normal but tubular sensitivity is impaired, usually due to distal tubular voltage defect (6). In type IV RTA, the mild renal disease could be because of systemic diseases like systemic lupus erythematosus [SLE], sickle cell disease, hepatitis, human immunodeficiency virüs (HIV) infections, lead nephropathy, analgesic nephropathy, chronic pyelonephritis, obstructive nephropathy, or some drugs (tacrolimus, trimetoprim, cyclosporin A and drugs affecting renin-alsosteron system) could be the cause of renal problem. Therefore it can be seen in situations other than diabetes mellitus (7–11). Because interstitial diseases cause more tubular damage, they are more likely to cause impaired renin production (e.g., in the juxtaglomerular apparatus) and therefore are more likely to compromise tubular potassium secretion in the distal nephron. Diabetic nephropathy, though primarily a glomerular disease, is an exception because it is associated with decreased renin production. Furthermore, in diabetic patients, the flow of potassium into the cells via beta-2 receptors is impaired due to insulin deficiency, thus negatively affecting extrarenal potassium homeostasis (12).

## Type IV RTA pathogenesis

Potassium filtered from the glomeruli is almost entirely reabsorbed by the proximal tubule and loop of Henle. Urinary excretion of potassium is mainly mediated by secretion of potassium into the lumen by the principal cell of the cortical collecting tubule. There are several factors that affect potassium excretion from the kidneys; 1) Sufficient sodium must reach the distal nephron, 2) Aldosterone must be present in the environment and provide sodium-potassium (Na-K) exchange, 3) There must be sufficient flow to remove the potassium secreted into the lumen (13, 14). Impaired renin production and release due to renal tubular damage, decreased aldosterone production as a result of adrenal dysfunction, or failure of principal cells to respond normally to aldosterone may impair potassium excretion and cause hyperkalemia. In true hyporeninemic hypoaldosteronism, atrophy of the juxtaglomerular apparatus may be present; this may be more prevalent in diabetics. Any combination of defects in these 3 steps may cause hyporeninemic hypoaldosteronism or RTA type IV. Indeed, as shown by Schambelan et al, all 3 factors may be present in some patients (15).

In patients with diabetes, various medications used for diabetes or concomitant diseases may affect renin release, aldosterone production, or tubular potassium excretion capacity. For example, beta-adrenergic blockade reduces renin release and leads to hyperkalemia in a given patient who is usually normokalemic. The drugs that may affect potassium levels are listed in **Table 1** (16–19).

**Table 1:** Drugs affecting serum potassium levels and may have role in type IV RTA and their mechanisms.

Mechanism	Drug groups and effects
Drugs that may contain potassium	Oral or intravenous penicillin, some herbal formulations
Drugs that inhibit or decrease renin	Beta blockers, including beta-1 selective ones, non-steroidal anti-inflammatory drugs, including cyclooxygenase-2 inhibitors, aliskren (direct renin inhibition)
Inhibitors of aldosterone	ACE inhibitors block formation of angiotensin II (a cofactor in aldosterone production), an effect similar to that of angiotensin II receptor blockers (ARBs); heparin interferes with adrenal gland aldosterone biosynthesis.
Inhibitors of potassium excretion	Spironolactone, eplerenone (direct competitive aldosterone inhibitors), triamterene and amiloride (inhibit the sodium channel necessary for potassium excretion), calcineurin inhibitors, including cyclosporine A and tacrolimus, may interfere with the aldosterone receptor, some herbal formulations
Drugs that impair potassium homeostasis	Nonselective beta blockers (and selective ones at higher doses) block beta2 -mediated potassium influx into cells, which is part of moment-to-moment potassium regulation; acute osmotic loads (e.g., from mannitol or radiocontrast material) cause osmotic efflux of water from cells, with convective drag of potassium—an effect mostly seen in diabetics, who lack the homeostatic protections of insulin release and an intact autonomic system

Hyperkalemia with hypoaldosteronism is usually associated with a mild metabolic acidosis with a normal anion gap. In type IV RTA, there is defect is in ammoniagenesis, but it still permits elaboration of acidic (pH < 5.3) urine. Hyperkalemia inhibits renal ammoniagenesis in several ways. Furthermore, it may produce acidosis by shifting protons from cells out to the extracellular space as homeostatic mechanisms attempt to buffer potassium by intracellular uptake.

Some genetic mutations, such as the NR3C2 gene and the RMND1 gene mutation, have also been studied as a cause of type IV RTA, but these are rare causes and usually occur in childhood (20, 21). There is also another situation with similar presentation, i.e. familial hyperkalemic hypertension (also known as pseudohypoaldosteronism type 2 or Gordon syndrome), which is associated with sodium and potassium retention due to defects in genes encoding serine-threonine kinases (WNK1 and WNK4) or ubiquitinating enzymes (CUL3 and KLCH3) expressed in the distal nephron and altering expression of the sodium-potassium co-transporter (22–24). Also in pseudohypoaldosteronism type 1, there is resistance to actions of aldosterone, but these situations also have clinical presentations without renal dysfunction or chronic diseases (25, 26).

In diabetic patients, presence of autonomic neuropathy and obesity could be an alarming finding to search tendency to type IV RTA. Because impaired neural control may cause decreased renin release and aldosteron production and neuropathic patients may have hyperkalemia (12, 27). In obese people, insulin resistance, oxidative stress and inflammation has role in decreased potassium uptake in cells and ammoniagenesis in the renal tubules, creating a metabolic environment relevant for type IV RTA (28).

## Incidence and diagnosis

The incidence of type IV RTA is difficult to estimate because it is usually asymptomatic and its incidence was reported as 3.8% of all hospital admissions in one single center (29). Patients are usually middle-aged to older, have chronic diseases such as diabetes, and are taking multiple medications.

Patients with type IV RTA generally have mild hyperkalemia, generally asymptomatic (30), but muscle weakness and arrythmias could be present. Normal anion gap and mild acidosis with serum bicarbonate levels in the ranging of 18–22 mEq/L may be observed. The patient can acidify urine (pH < 5.3) which distinguishes type IV RTA from type I RTA. Acid urine pH differs from type 1 distal RTA, reflecting the preservation of distal acidification capacity shown when the concentration of urine buffer (NH4+) for excreted acid valences is decreased.

The impact of hypoaldosteronism (absolute and/or relative) at a tubular level may be assessed indirectly through the transtubular potassium gradient (TTKG). The TTKG reflects the distal tubular potassium secretion ability (in the collecting duct) and is calculated as follows:


TTKG=(Urine Potassium × Serum Osmolality)

(Serum Potassium × Urine Osmolality)

In the presence of hyperkalemia, the TTKG is physiologically expected to reach values above 7, reflecting compensatory hyperaldosteronism. In hyperkalemic RTA, the TTKG is lower than 7. TTKG variations with therapy (for example, with fludrocortisone) help differentiate between aldosterone deficiency and resistance:

- Aldosterone deficiency: early normalization (2-4h) of the TTKG after physiological doses of fludrocortisone (0.1mg/day).
- Aldosterone resistance: late normalization (>24h) of the TTKG after supraphysiological doses of fludrocortisone (> 0.1mg/day).

Some authors advice use of urine anion gap or urine osmolal gap (30). In a healthy patient, high potassium intake is followed by a high urinary potassium excretion rate; in the presence of hyperkalemia, low urinary potassium is first evidence of inadequate renal potassium excretion.

Urine anion gap may not be very reliable in the presence of urine Na+ levels below 25 mmol/L (frequently seen in cases of prerenal acute kidney injury), increased excretion of non-measured anions (ketoacidosis, bicarbonate therapy, D-lactacidemia, poisoning by toluene or paracetamol), and during the neonatal period (a stage in which non-measured anions are excreted in large quantities) (2).

## Treatment

Treatment strategies focus on correction of potassium levels and acidosis. First step in treatment is elimination of medications that may cause hyperkalemia and to focus on the history of renal disease. Other important point is dietary precautions with potassium restriction. Since glucocorticoid excretion is normal, they do not have sign of adrenal insufficiency, but they are generally hypertensive. If potassium is higher than 6.0 mEq/L electrocardiogram should be seen and emergency treatment of hyperkalemia should start.

The measurement of renin and aldosteron levels are not useful for treatment and not cost-effective, also their standardized measurements are difficult due to their changes with position, etc. Therefore unless there is clinical uncertainty it is not necessary to measure renin and aldosterone.

For further increase in potassium excretion, both loop and thiazide diuretics could be used in treatment. Volume depletion and alkalosis may be adverse effects due to overtreatment. Sodium bicarbonate corrects acidosis by increasing distal delivery of carbonate anion, increases potassium excretion. Fludrocortisone at 0.1-0.3 mg/day doses may provide kalliuresis with mineralocorticoid effect, but may increase fluid overload and cause hypertension, also has some glucocorticoid metabolic effects, difficult to manage in patients with chronic diseases such as heart failure and uncontrolled diabetes. Therefore, treatment with fludrocortisone is typically reserved for cases refractory to diuretic therapy (loop or thiazides), low potassium diets, and ion-exchange resins (30). Potassium- binding resins could be used to decrease potassium. Generally a stable potassium level below 5.5 mEq/L is acceptable for outpatient follow-up.

## Conclusion

Diabetes is chronic disease with multiple complications and world-wide increasing prevalance (31). Diabetic people generally have kidney failure and electrolyte imbalances secondary to impaired renal function, but hyperkalemia with normal kidney tests and hyporeninemic hypoaldosteronism is a frequently underdiagnosed medical condition. It is more prevalent in individuals with diabetes with mild to moderate renal impairment. Since these patients use many medications due to their accompanying diseases, the pharmacological causes that trigger hyperkalemia should be investigated first. Also, treatment strategies focus on normalizing the potassium levels and protecting kidney functions. Type IV RTA may be present before renal dysfunction becomes evident, and clinicians should be more careful in monitoring patients with diabetes, as most patients with diabetes require renin-angiotensin inhibitors or beta-blockers because of cardiovascular problems and hypertension.
